# The Clinical Relevance of IL-17-Producing CD4+CD161+ Cell and Its Subpopulations in Primary Sjögren's Syndrome

**DOI:** 10.1155/2015/307453

**Published:** 2015-09-08

**Authors:** Linbo Li, Jing He, Lei Zhu, Yuqin Yang, Yuebo Jin, Rulin Jia, Xu Liu, Yanying Liu, Xiaolin Sun, Zhanguo Li

**Affiliations:** Department of Rheumatology & Immunology, Clinical Immunology Center, Peking University People's Hospital, 11 Xizhimen South Street, Beijing 100044, China

## Abstract

*Objective*. Th17 cells have been demonstrated to play an important role in the onset and development of primary Sjögren's syndrome (pSS). In this study, we evaluated the expansion and clinical significance of circulating CD4+CD161+ T cell and its “effector” (CD4+CD25−CD161+ T cell) and “regulatory” (CD4+CD25+CD161+ T cell) subpopulations.* Methods*. Fifty-eight pSS patients and 16 healthy controls (HCs) were recruited in our study. The cell populations and intracellular IL-17 expression were analyzed by flow cytometry. The disease activity was evaluated by the EULAR-SS Disease Activity Index (ESSDAI). Autoantibodies were measured by ELISA or indirect immunofluorescence assay.* Results*. The CD161+ T cell fractions showed higher proportions of IL-17-producing cells. The frequencies of the overall CD4+CD161+ T cell population and its effector subset were positively correlated with disease activity parameters and more severe disease manifestations. A significant elevation of the CD4+CD25+CD161+ T cell subpopulation was observed in the peripheral blood of pSS patients compared to HCs and this subset showed decreased regulatory functions compared with the CD4+CD25+CD161− population.* Conclusion*. Circulating CD4+CD161+ T cell populations associated with pSS disease activity and severity. These cells might be involved in the development of pSS and could be potential therapeutic targets in the treatment of pSS.

## 1. Introduction

Primary Sjögren's syndrome (pSS) is a systemic autoimmune disease characterized by autoimmune damage of salivary glands (SG) and lacrimal glands that leads to dry-mouth and dry-eye symptoms. At least one-third of patients developed extraglandular cutaneous, pulmonary, renal, or neurological manifestations, which severely affected the quality of life and could cause lethal consequences [1, 2]. Comprehensive effects of immunological, genetical, and environmental factors are involved in the pathogenesis of pSS, whose exact etiology remains elusive.

It has been shown that various immune cell populations such as macrophages, dendritic cells, T cells, and B cells are involved in the pathogenesis of pSS [3–5]. A predominant presence of CD4+ T cells in inflammatory infiltrates in SG implied that T cell subsets might contribute to glandular damage in pSS [6, 7]. Th17 cells, which are characterized by the production of proinflammatory cytokine IL-17, have been proved to be involved in inflammation, autoimmunity, and glandular tissue damage in pSS [8–10]. It has been found that adoptive transfer of Th17 cells into the inducible IL-17A KO mice which are resistant to the induction of pSS could rapidly acquire a pSS profile [11]. In patients with pSS, presence of Th17 cells has been observed in inflamed tissues [12, 13]. In the salivary glands of pSS patients, Th17 cells were identified as the predominant infiltrating T cells [12].

CD161, also known as KLRB1 (killer cell lectin-like receptor subfamily B, member 1), interacts with the ceramide-generating enzyme acid sphingomyelinase and is involved in cellular signaling and activation [14, 15]. Recently, Cosmi et al. [16, 17] have shown that CD161 is the marker of all human IL-17-producing T cell subsets and human Th17 cells originate from the CD4+CD161+ T cell subset. Subsequent studies have highlighted a pathogenic role of CD4+CD161+ T cells in rheumatoid arthritis (RA), juvenile idiopathic arthritis (JIA), Crohn's disease-associated perianal fistulas, and asthma [18–21]. The frequency of circulating CD4+CD161+ T cells increases in RA patients and is positively correlated with the 28-joint disease activity score (DAS28), CRP, VAS score, and so forth [19]. The accumulation of CD161+ T lymphocytes was associated with CD patients with complex fistulizing perianal disease [21].

According to CD25 expression, CD4+CD161+ T cells can be further divided into the “regulatory” (CD4+CD25+CD161+ T cell) and “effector” (CD4+CD25−CD161+ T cell) subpopulations. Recent studies in inflammatory arthritis have identified CD4+CD25+CD161+ T cells as the IL-17-producing Th17-like Treg cells [22, 23], which showed decreased suppressive activity and might contribute to autoimmunity in inflammatory sites. The CD4+CD25−CD161+ T effector cells do not possess suppressive function and might be pathogenic in autoimmune conditions due to their IL-17 production; however, there is no clinical and experimental data directly addressing this point.

In pSS, the clinical relevance of circulating CD4+CD161+ T cells and their subpopulations remain elusive. In this study, we evaluated the production of IL-17 of circulating CD4+CD161+ T cells in pSS and determined the levels of circulating CD4+CD161+ T cells and their two subsets (CD4+CD25+CD161+ T and CD4+CD25−CD161+ T) in pSS patients. We evaluated the levels of these cell populations and analyzed the clinical significance of these cell subsets. Our study showed that CD4+CD161+ T cell subsets are positively correlated with the disease activity and severity of pSS and may be potential therapeutic targets for the treatment of pSS.

## 2. Materials and Methods

### 2.1. Patients and Healthy Controls

A total of 58 pSS patients of the Department of Rheumatology and Immunology, Peking University People's Hospital, were recruited in the study. All patients diagnosed as pSS fulfilled the 2002 American-European Consensus Group Criteria [1] and individuals combined with other rheumatic diseases were excluded. Disease activity was evaluated by ESSDAI. 16 healthy controls were collected from health physical examination personnel at the same hospital. There were no significant differences in the ages or sex ratios between the patient and control groups. This study was approved by the ethics committee of Peking University People's Hospital. Written consent was provided by all the patients recruited to donate their blood samples and clinical information in this study.

### 2.2. Flow Cytometry Analysis

Peripheral blood mononuclear cells (PBMCs) were isolated by Ficoll-Hypaque density gradient centrifugation. For surface staining, PBMCs were stained for 30 min in the dark at 4°C with the monoclonal antibodies PE-CF594 mouse anti-human CD4 (BD Biosciences, San Diego, CA, USA), PE anti-human CD25 (BioLegend, San Diego, CA, USA), FITC anti-human CD161 (BioLegend), 7AAD (BD Pharmingen, San Diego, CA, USA), and CD45RO PE-Cy7 (BD Biosciences) or with FITC Mouse IgG1 *κ* isotype control (BioLegend) and Mouse IgG1 *κ* isotype control PE (eBioscience, San Diego, CA, USA). For intracellular staining of cytokines, incubate PBMCs in RPMI 1640 medium (Gibco, Life Technologies, Shanghai, China) in 5% CO_2_ at 37°C, then stimulate these cells for 5 h, PBMCs were stimulated for 5 h with 50 ng/mL phorbol 12-myristate 13-acetate (PMA; Sigma-Aldrich, Steinheim, Germany) and 1 *μ*g/mL ionomycin (BD Pharmingen) in the presence of 10 *μ*g/mL Brefeldin-A (BFA; BioLegend) and subsequently fixed and permeabilized by Foxp3/Transcription Factor Staining Buffer Set (eBioscience). Then the cells were stained for 30 min away from the light at 4°C with anti-human IL-17A APC (eBioscience) or Mouse IgG1 *κ* Isotype Control APC (eBioscience).

### 2.3. T Cell Sorting and Suppression Assay

CD4+CD25+ and CD4+CD25− T cells were enriched from PBMCs by magnetic cell sorting (StemCell Technologies, Vancouver, BC, Canada) and then stained with FITC anti-human CD161 (BioLegend) and sorted further into the CD161+ and CD161− fractions using a BD Aria II flow cytometer. The CD4+CD25+CD161+ or CD4+CD25−CD161+ T cells or CD4+CD25− T cells were cocultured with effector T cells (Teff, CD4+CD25− T) from the third-party healthy donors stained with CFSE (Invitrogen, CA, USA) together with the Treg Suppression Inspector beads (Miltenyi Biotec GmbH, Bergisch Gladbach, Germany). Their proliferation in 7 days was evaluated by flow cytometry.

### 2.4. Clinical Data Analysis

The following features of pSS were included in this study: xerostomia, xerophthalmia, parotid gland enlargement, swollen and/or tender joints, interstitial lung diseases, anaemia (Hb < 115 g/L), leucopenia (white blood cell count <3,500/*μ*L), and thrombocytopenia (platelet count <125,000/*μ*L). All patients underwent extensive serological examinations, including tests of antinuclear antibody (ANA), anti-Ro/SSA antibody (anti-SSA), anti-La/SSB antibody (anti-SSB), anti-*α*-Fodrin antibody, rheumatoid factor (RF), complement component C3, complement component C4, erythrocyte sedimentation rate (ESR), C-reactive protein (CRP), immunoglobulin A (IgA), immunoglobulin G (IgG), immunoglobulin M (IgM), and *γ*-globulin. Anti-*α*-Fodrin antibody, C3, C4, IgA, IgG, and IgM were tested by ELISA with normal ranges of 0–18 U/mL, 0.79–1.52 G/L, 0.16–0.38 G/L, 0.82–4.53 G/L, 7.2–16.8 G/L, and 0.46–3.04 G/L. Anti-SSA and anit-SSB were measured by ELISA, ANA was measured by indirect immunofluorescence, and RF was measured by immune turbidimetry. Positive RFs were defined as values equal or more than 20 IU/mL. CRP was examined by immunonephelometry method and values equal or more than 8 mg/L were considered positive. The fractional percentage of *γ*-globulin was checked by performing serum protein electrophoresis with a normal range of 11.1–18.8%.

The disease activity was evaluated using ESSDAI, a clinical index of disease activity measurement based on the assessment of 12 domains (constitutional, lymphadenopathy, glandular, articular, cutaneous, pulmonary, renal, muscular, peripheral nervous system, central nervous system, hematological, and biological).

### 2.5. Statistical Analysis

SPSS 13.0 for windows and GraphPad Prism 5 were used to analyze the data. Data were presented as mean ± standard deviation and statistical significance between two groups was assessed with the nonparametric Mann-Whitney test, paired *t*-test, *χ* ^2^ test, logistic regression, and analysis of covariance (ANCOVA). Spearman's rank correlation coefficient was applied to calculate the correlations. A value of *p* < 0.05 was considered to be significant. The cut-off values of T cell subsets were determined by receiver operating characteristics (ROC) curve.

## 3. Results

### 3.1. Characteristics of pSS Patients

Demographic, clinical, and laboratory characteristics of pSS patients and healthy controls are shown in Table 1. 58 pSS patients and 16 healthy controls with matched age and gender were recruited in this study (age: 57.84 ± 13.01 versus 51.59 ± 18.58, *p* = 0.158; gender: *p* = 0.524). The pSS patients had a mean disease duration of 7.51 years ranging from 1 to 30.67 and the mean ESSDAI score of these patients was 3.86 ranging from 1 to 9 (Table 1).

### 3.2. The Phenotypic Characteristics of CD4+CD161+ T Cells in pSS Patients

**Figure 1:**
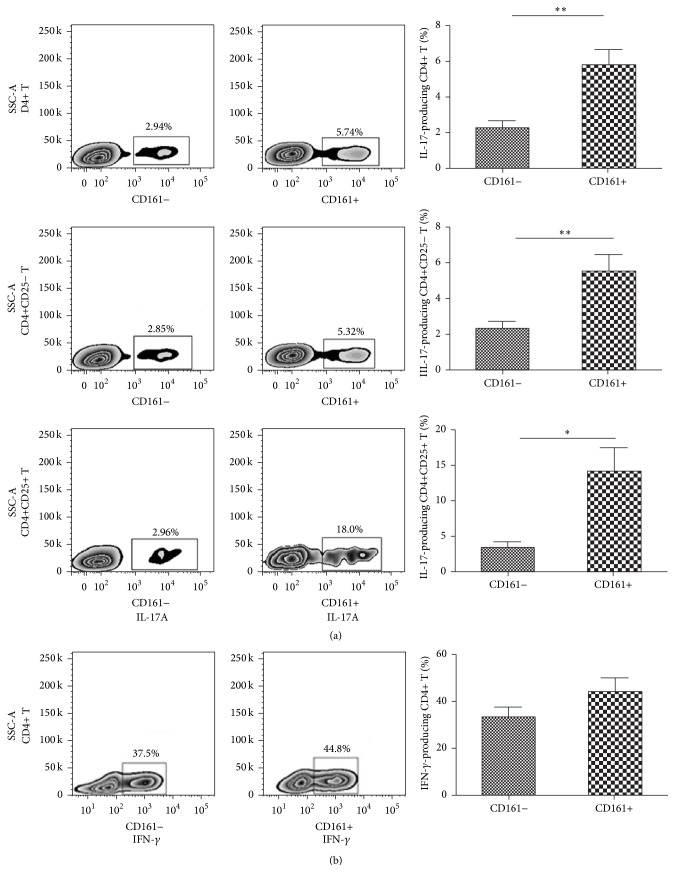
IL-17 and IFN-*γ* production of the CD4+CD161+ T cell subsets in pSS. Representative flow cytometric plots of intracellular IL-17 or IFN-*γ* production in CD161+ or CD161− fraction of different T cell subsets were shown. Percentages of IL-17-producing or IFN-*γ*-producing cells in CD161+ and CD161− fractions in pSS patients (*n* = 6) were compared by paired *t*-test. ^*∗*^ *p* < 0.05, ^*∗∗*^ *p* < 0.01.

We assessed the intracellular IL-17 expression in circulating CD4+ T cells of pSS patients. Both of the CD161+ and CD161− subsets of CD4+ T cells expressed IL-17, while the percentage of IL-17-producing cells was significantly higher in the CD161+ fraction than in CD161− one (5.76 ± 2.21 versus 2.24 ± 0.94, *p* = 0.0025, Figure 1(a)). For the effector or regulatory subpopulation of CD4+CD161+ T cells, higher frequency of IL-17-producing cells was also detected when compared with their CD4+CD161− counterpart in pSS patients (5.52 ± 2.28 versus 2.30 ± 0.99, *p* = 0.0058; 14.15 ± 7.95 versus 3.33 ± 1.96, *p* = 0.0169, Figure 1(a)). The IFN-*γ* production of CD4+CD161+ T cells was also evaluated. Similar to IL-17, IFN-*γ* was expressed by both of the CD161+ and CD161− subsets. Although IFN-*γ* expression was increased in CD161+ subset compared to the CD161− subsets, there was no statistical significance between the IFN-*γ* production level of these two subsets (28.88 ± 9.04 versus 43.02 ± 15.67, *p* > 0.05, Figure 1(b)).

We also compared IL-17 and IFN-gamma production of CD4+CD161+ T cells in pSS patients and healthy controls. The IL-17 expression in both CD4+CD161+ and CD4+CD161− T cells was higher in pSS patients than in healthy controls, but it did not reach statistical significance (IL-17, pSS versus HC: CD161− 2.53 ± 1.29 versus 1.86 ± 1.39, *p* = 0.4641; CD161+ 5.29 ± 2.37 versus 3.98 ± 2.61, *p* = 0.4318). For IFN-gamma production we did not detect any difference between pSS and HC patients in these two T cell subsets (IFN-r, pSS versus HC: CD161− 28.78 ± 10.23 versus 29.18 ± 8.64, *p* = 1.0000; CD161+ 47.82 ± 11.80 versus 43.18 ± 15.59, *p* = 0.8413).

**Figure 2:**
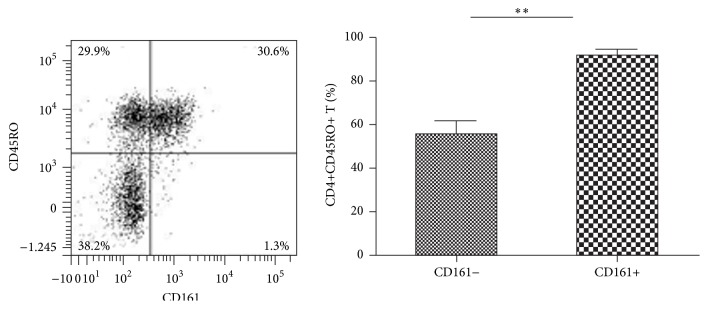
Most CD4+CD161+ T cells are CD45RO+. Representative flow cytometric plots of CD45RO expression on CD161+ or CD161− fraction of CD4+ T cell subset were shown. Percentages of CD4+CD45RO+ T cells in CD161+ and CD161− fractions in pSS patients (*n* = 6) were compared by paired *t*-test. ^*∗∗*^ *p* < 0.01.

According to the study by Cosmi et al., Th17 cells mainly originated from the CD4+CD161+ T cell precursors [16]. To further identify whether the CD4+CD161+ T cell subset actively producing IL-17 in this study has already attained the memory phenotype or not, we analyzed the cell-surface expression of CD45RO on these cells from pSS patients. Both of the CD161+ and CD161− subsets expressed CD45RO, while most of the CD4+CD161+ T cells were CD45RO+ cells, compared with the CD161− ones (92.74 ± 4.28 versus 55.98 ± 12.80, *p* = 0.0079, Figure 2), which clearly showed that nearly all the CD4+CD161+ T cells were memory T cells.

### 3.3. Levels of Circulating CD4+CD161+ T Cell Subsets in pSS Patients

**Figure 3:**
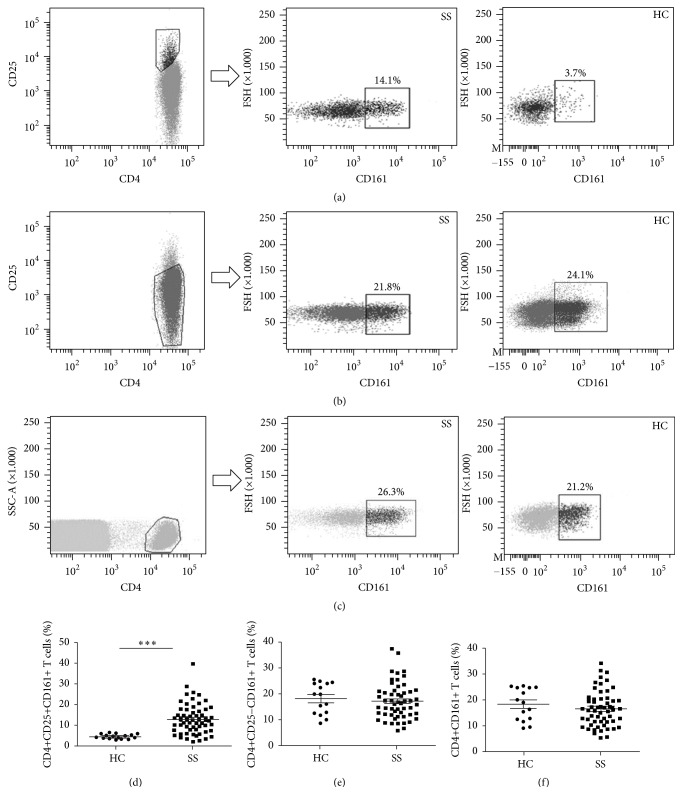
FACS analysis of circulating CD4+CD161+ T cell subsets. Representative data of CD4+CD25+CD161+ T (a), CD4+CD25−CD161+ T (b), and CD4+CD161+ T (c) from peripheral blood of patients with pSS (*n* = 58) and HCs (*n* = 16) were shown and percentages of circulating CD4+CD25+CD161+ T in CD4+CD25+ T (d), CD4+CD25−CD161+ T in CD4+CD25− T (e), and CD4+CD161+ T in CD4+ T (f) in pSS patients and HCs were compared by Mann-Whitney *U* test. ^*∗∗∗*^ *p* < 0.0001.

We evaluated the levels of CD4+CD161+ T cells and their two subpopulations in PBMCs of pSS patients and HCs by flow cytometry (Figures 3(a)–3(c)). Compared with HCs, the pSS patients showed a significant increase of circulating CD4+CD25+CD161+ T cell subset (13.04 ± 7.32 versus 4.60 ± 1.05, *p* < 0.0001, Figure 3(d)), while the levels of CD4+CD25−CD161+ T and the overall CD4+CD161+ T in PBMCs were not significantly different between the pSS patients and HCs (both *p* > 0.05, Figures 3(e) and 3(f)).

### 3.4. Correlation between Clinical and Laboratory Characteristics in pSS Patients and CD4+CD161+ T Cell Subsets

Three cut-off values were determined by ROC analysis to distinguish pSS patients with overelevated CD161+ T cell subsets from those bearing normal CD161+ T cell subset levels comparable to the healthy controls (6.70% for CD4+CD25+CD161+ T cells, 17.79% for CD4+CD25−CD161+ T cells, and 17.66% for overall CD4+CD161+ T cells; Table 2). According to the cut-off value, CD4+CD161+ T cells and their two subpopulations were divided into the elevated group and the normal group, respectively.

**Figure 4:**
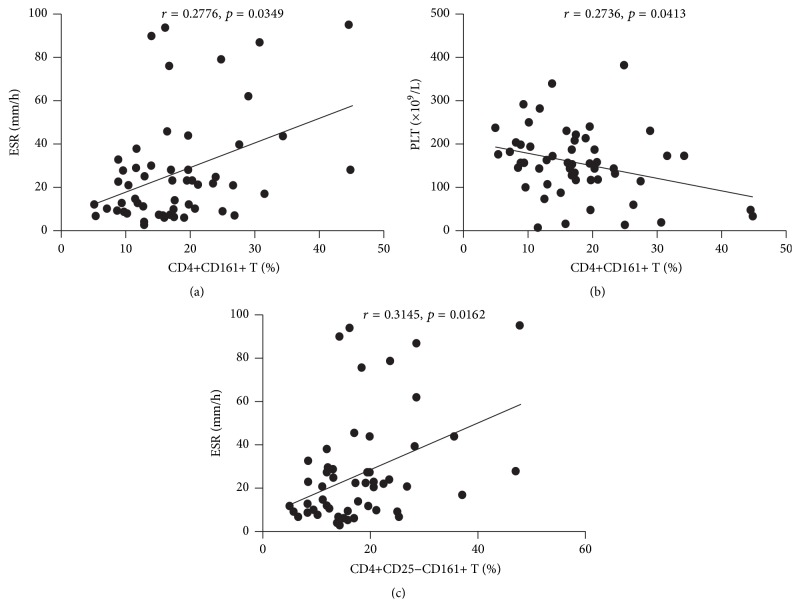
Correlations of circulating CD4+CD161+ T cell subsets in pSS with laboratory values. CD4+CD161+ T cells were positively correlated with ESR (a) or platelet reduction (b). CD4+CD25−CD161+ T cells were positively correlated with ESR (c).

**Figure 5:**
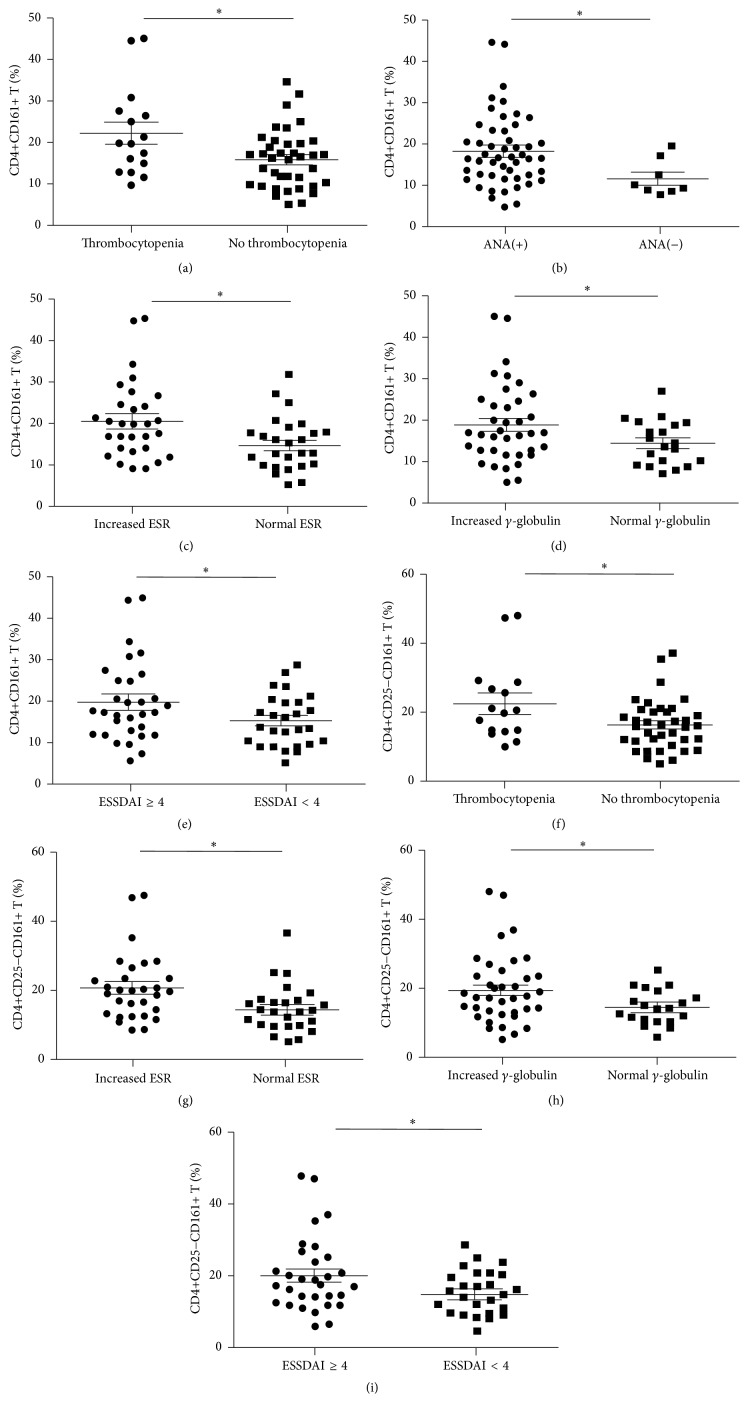
Percentages of CD4+CD161+ T (a to e) and CD4+CD25−CD161+ T (f to i) cells according to the disease features. ANA: antinuclear antibody; ESR: erythrocyte sedimentation rate; ESSDAI: the EULAR-SS Disease Activity Index. ^*∗*^ *p* < 0.05.

For the overall CD4+CD161+ T cells, it was found that their level was associated with increased ESR (*χ* ^2^ = 5,346, *p* = 0.021) even with further multivariate analysis (*p* = 0.020). We subsequently analyzed the correlation between CD4+CD161+ T cells and laboratory features of pSS. As shown in Table 3 and Figure 4, CD4+CD161+ T was positively correlated with ESR (*r* = 0.2776, *p* = 0.0349) or platelet reduction (*r* = −0.2736, *p* = 0.0413). This subset also showed a tendency of correlation with anti-SSB levels (*r* = 0.2498, *p* = 0.0586) at the border of statistical significance. The percentages of CD4+CD161+ T cells were then compared between patient groups with or without autoimmune clinical and laboratory features. Patients with thrombocytopenia, ANA positivity, increased ESR, elevated *γ*-globulin, or ESSDAI ≥ 4 showed higher proportions of CD4+CD161+ T (22.21 ± 10.69% versus 15.95 ± 6.95%, *p* = 0.013; 18.56 ± 8.71% versus 12.03 ± 4.33%, *p* = 0.043; 20.53 ± 9.35% versus 14.86 ± 6.48%, *p* = 0.012; 19.20 ± 9.49% versus 14.73 ± 5.36%, *p* = 0.026; 19.71 ± 9.77% versus 15.30 ± 6.18%, *p* = 0.049; Table 4, Figures 5(a)–5(e)). Further multivariate analysis still showed that patients with increased ESR had significantly higher proportion of CD4+CD161+ T cell (*p* = 0.005), which implicated the proinflammatory role of this cell subset.

The clinical relevance of the effector and regulatory subsets of CD4+CD161+ T cells was evaluated by the same analysis. Although the regulatory subset (CD4+CD25+CD161+ T cells) of CD4+CD161+ T cells was significantly elevated in pSS patients, the clinical relevance analysis did not show any significant association of this subset with the clinical and laboratory features of pSS (Tables 2, 3, and 4). The effector subset (CD4+CD25−CD161+ T cell) showed remarkably similar clinical relevance pattern to the whole CD4+CD161+ T cells. Associations were also found between the effector subset and increased ESR (*p* = 0.010). Furthermore, a positive correlation was verified between this subpopulation and ESR (*r* = 0.3145, *p* = 0.0162). A tendency of mild positive correlation between this subset and anti-SSB levels (*r* = 0.2562, *p* = 0.0523) or platelet reduction (*r* = −0.2562, *p* = 0.0566) was also observed (Table 3, Figure 4). Patients with thrombocytopenia, increased ESR, elevated *γ*-globulin, or ESSDAI ≥ 4 possessed higher proportions of effector subset than those without the characteristic above in univariate analysis (22.43 ± 11.37% versus 16.09 ± 7.30%, *p* = 0.018; 20.83 ± 9.66% versus 14.74 ± 6.96%, *p* = 0.010; 19.54 ± 10.00% versus 14.47 ± 5.07%, *p* = 0.013; 20.11 ± 10.37% versus 15.13 ± 6.02%, *p* = 0.032; Table 4, Figures 5(f)–5(i)). However, no statistical significance was observed when further multivariate analysis was performed.

### 3.5. Impaired Regulatory Functions of CD4+CD25+CD161+ T Cells in pSS Patients

**Figure 6:**
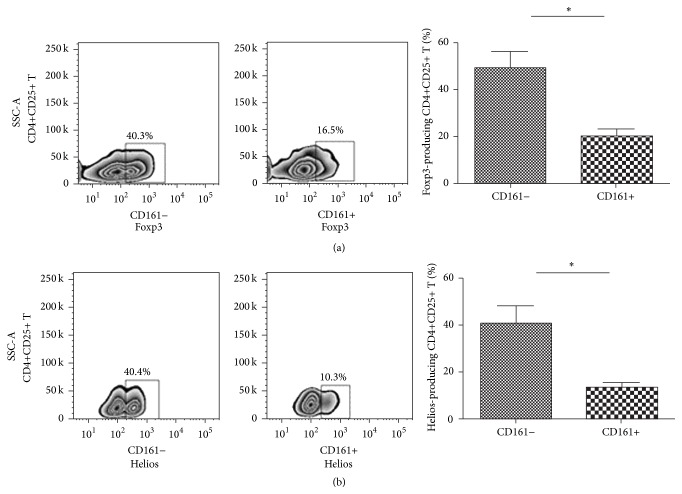
Foxp3 and Helios expression of the CD4+CD25+CD161+ T cell subsets in pSS. Representative flow cytometric plots of transcription factor production in CD161+ or CD161− fraction of different T cell subsets were shown. Percentages of Foxp3-producing or Helios-producing cells in CD161+ and CD161− fractions in pSS patients (*n* = 6) were compared by paired *t*-test. ^*∗*^ *p* < 0.05.

Foxp3 and Helios are key transcription factors of regulatory T cell function and development. Foxp3 is the master regulator of the Treg lineage, and Helios is the specific transcription factor and marker for natural Tregs which develop in thymus and keep a more stable regulatory phenotype than the induced Tregs developing in the peripheral blood. The expression of the two functional regulators was evaluated in both CD4+CD25+CD161+ and CD4+CD25+CD161− T cells of pSS patients. It was showed that CD4+CD25+CD161+ T cells expressed significantly lower Foxp3 and Helios than CD4+CD25+CD161− subpopulation (49.20 ± 14.23 versus 19.75 ± 6.77, 41.04 ± 15.33 versus 13.17 ± 6.64, *p* < 0.05, Figures 6(a) and 6(b)), which implicated that the CD4+CD25+CD161+ T cells might process decrease of immunoregulatory functions compared with CD4+CD25+CD161− T cells.

We further compared the expression levels of Foxp3 and Helios in CD4+CD25+CD161+ and CD4+CD25+CD161− T cells from pSS patients and healthy controls (Supplementary Figures  1 and 2; see Supplementary Material available online at http://dx.doi.org/10.1155/2015/307453) and found that in comparison to their CD161+ or CD161− counterparts from healthy controls, the CD4+CD25+CD161+ or CD4+CD25+CD161− T cells from pSS patients had significantly elevated Foxp3 or Helios expression levels (Supplementary Figures  1 and 2). This finding suggested an increase of Treg frequency took place in pSS conditions, which might reflect the negative feedback to T cell responses to downregulate the autoimmunity.

**Figure 7:**
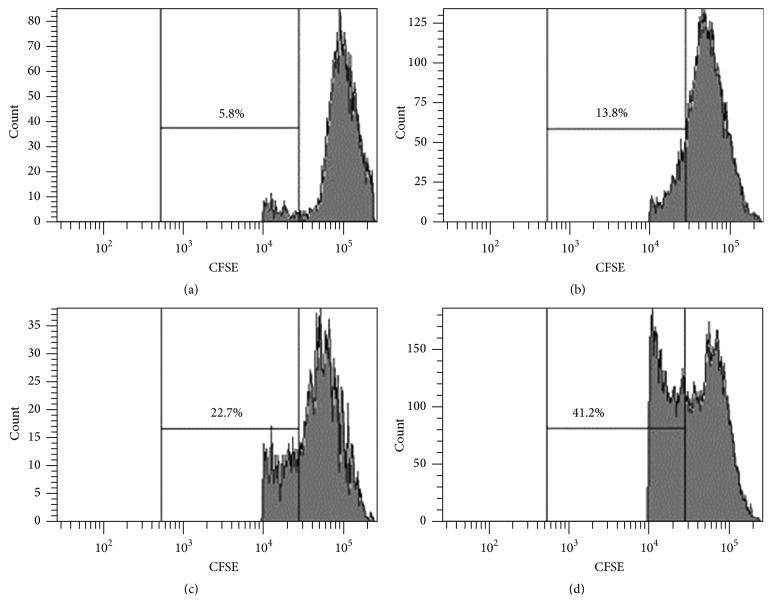
Compared with CD4+CD25−CD161+ T cells, the CD4+CD25+CD161+ T subset from peripheral blood of pSS patients showed impaired suppression activity on CD4+CD25− Teff from HC. (a) Unstimulated control; (b) CD4+CD25+CD161− T and Teff; (c) CD4+CD25+CD161+ T and Teff; (d) CD4+CD25− T and Teff. This result was representative of three independent experiments.

To directly assess the regulatory function of these T cell subsets, in vitro suppression assay was performed. Although CD4+CD25+CD161+ T cells from pSS patients retained suppressive activity on CD4+CD25− effector T cell proliferation, they showed decreased suppression on CD4+CD25− effector T cell proliferation compared with the CD4+CD25+CD161− T cells, which confirmed the impaired regulatory function of this inflammatory cytokine producing Treg subset (Figure 7).

## 4. Discussion

Th17 cells have been proved to contribute to autoimmune responses and tissue destruction in rheumatic diseases [24, 25]. Recent studies have highlighted the pathogenic role of Th17 cells in pSS [26–28]. IL-17 might launch local inflammatory responses and further induce production of a variety of proinflammatory cytokines in pSS. Apart from this, Th17 cells have been proved to be involved in pathogenesis of pSS by producing IL21 [29–32] and IL22 [27] and upregulate matrix metalloproteinases (MMPs) [33, 34]. Circulating IL22 was significantly elevated in pSS and showed significant correlations with major characteristics such as xerostomia, anti-SSB, rheumatoid factor, and hypergammaglobulinemia [35]. Th17 cells are also highly effective B cell helpers not only inducing a strong proliferative response of B cells in vitro but also triggering antibody production with class switch recombination in vivo, via the B cell stimulating functions of IL-17 and IL21 [36]. As the IL-17-producing cells, clinical relevance of CD4+CD161+ T cells has been addressed in several rheumatic diseases. Cosmi et al. have found the frequencies of CD4+CD161+ cells in the JIA synovial fluid are positively correlated with ESR and levels of CRP [18]. Maggi et al. have proved that CD4+CD161+ T lymphocytes infiltrate Crohn's disease-associated perianal fistulas and are reduced by anti-TNF*α* local therapy [21].

Besides the roles of pathogenic cell subsets such as Th17 in pSS development, abnormal cytokine production in pSS has been investigated for years. Early studies revealed that IFN-*γ* elevation will cause epithelium destruction in targeted glands in pSS, and pSS was considered as a Th1 driven disease for a long time [37]. The elevation of BAFF was also proved to be one of the main reasons for the overactivation of B cells in pSS [38, 39]. Recent studies identified the high expression of Th17 related cytokines in peripheral blood or salivary glands from pSS patients, which included IL-17, IL-6, IL-21, IL-22, and IL-23 [26–28, 37]. The concurrent presence of these Th17 cytokines stabilized Th17 phenotype and will trigger the downstream inflammatory events.

In this study, we found that most of the CD4+CD161+ T cells are CD45RO+ memory T cells. This suggested that CD4+CD161+ T cells had gone through antigen activation in pSS and could respond to the antigens released by target organs of pSS. This study examined the intracellular IL-17 and IFN-*γ* expression in pSS patients and verified that CD161+ subpopulation was more capable of producing IL-17 than its CD161− counterparts. This observation was in agreement with previous studies that CD161+ T cells contain the majority of human Th17 cells [18–21]. It was also shown that CD4+CD161+ T cells expressed more IFN-*γ* than the CD4+CD161− T cells, though the difference did not reach statistical significance, which was in accordance with the previous observation that CD161+ T produces significantly more IFN-*γ* than the CD161− subset in asthma [40]. Since both IFN-*γ* and IL-17 could promote the development of pSS [13, 28, 37, 41], CD4+CD161+ T cells would contribute to pSS pathogenesis at least by expressing these proinflammatory cytokines. Besides proinflammatory cytokine expression, the migratory capacity of CD161+ T cells had been reported by previous studies [42]. These cells could migrate to inflammatory tissues due to expression of specific chemokines like lectin-like transcript 1 or chemokine receptors such as CXCR16 or CD161 itself [43]. Once the CD161+ T cells located in the inflammatory tissues, they may function like Th17 or Th1 to enhance autoimmunity or take part in tissue damage by direct interactions with local tissue components. A recent study also reported the CD161+ Th17 lineage cells are resistant to regulatory T cell-mediated suppression in the context of autoimmunity [44]. The exact pathogenic roles they play in targeted tissues are still elusive now and need to be exploited in the future studies.

We further revealed the clinical significance of CD4+CD161+ T cell subsets in pSS for the first time, especially focusing on the correlation between these subsets and pSS disease activity parameters. The circulating CD4+CD161+ T cell levels were positively correlated with ESR, thrombocytopenia, and anti-SSB in pSS. Patients with ANA or elevated *γ*-globulin also showed higher levels of CD4+CD161+ T cells. More circulating CD4+CD161+ T cells were found in patients with higher disease activity (ESSDAI ≥ 4) than patients with lower disease activity. Further multivariate analysis confirmed the association of CD4+CD161+ T cells with inflammatory markers like increased ESR. These results suggest that IL-17-producing CD4+CD161+ T cells might play a role in the inflammation development and B cell activation in pSS, at least by the effects of IL-17, though no significant difference of their levels was observed between patients with pSS and HCs.

CD25 has been identified as the key surface marker of regulatory T cells (Tregs). CD4+CD25^bright^ Treg cells play anti-inflammatory and immunosuppressive roles in pSS [45]. Our recent finding has reported that CD161 expression defined an IL-17-producing Treg subset which might be pathogenic in inflammatory articular sites in rheumatoid arthritis [22]. Therefore, in this study we further analyzed two subsets of CD4+CD161+ T cells (we named them effector and regulatory subpopulation), which were characterized by the absence or presence of CD25 expression on cell surfaces. In both two fractions, the frequencies of CD161+ subsets were compared between pSS patients and HCs. CD4+CD25−CD161+ T, the effector subpopulation, the majority of CD4+CD161+ T cells, showed similar clinical relevance to the overall CD4+CD161+ T cells in pSS, either in distribution in peripheral blood between patients and HCs or in correlations with disease activity parameters. CD4+CD25+CD161+ T cells, the regulatory subset of CD4+CD161+ T cells, significantly increased in pSS patients and the ratio of IL-17-producing cells of this subset was much higher than that in the overall CD4+CD161+ T cells (14.15 ± 7.95% versus 5.76 ± 2.21%, *p* = 0.034) and CD4+CD25−CD161+ subset (14.15 ± 7.95% versus 5.52 ± 2.28%, *p* = 0.032), which might implicate that abnormal Treg functions could take place in pSS. The regulatory function of the “regulatory” subset of CD4+CD161+ T cells was also evaluated in this study. CD4+CD25+CD161+ T cells expressed significant lower Foxp3 and Helios than the CD4+CD25+CD161− subset, and in vitro suppression assay directly showed decreased suppression activity of the CD161+ Treg subset. These results indicated that CD4+CD25+CD161+ T cells processed impaired regulatory function when they actively produced more proinflammatory cytokines like IL-17, which implicated that they might play pathogenic roles in pSS. However, no significant association between this subset and clinical features was identified. This may be caused by the relatively smaller percentages of this cell subset. Since the CD4+CD25+CD161+ T cells are rare, their overall contribution to pSS disease development might not be so obvious. On the other hand, the limited sample number recruited in this study might be another reason for the lack of statistical significance. Further studies are needed to elucidate the exact roles of this IL-17-producing Treg subset in pSS.

## 5. Conclusions

In this study, we determined the levels of circulating CD4+CD161+ T cells and their “regulatory” (CD4+CD25+CD161+ T cell) and “effector” (CD4+CD25−CD161+ T cell) subpopulations in patients with pSS and analyzed the clinical significance of the CD4+CD161+ T cell subsets in pSS for the first time. This study showed that CD4+CD161+ T cell subpopulations were more capable of producing IL-17 than their CD161− counterparts. CD4+CD25+CD161+ T cells, the regulatory subset of CD4+CD161+ T cells, significantly increased in pSS patients, which showed abnormality in Treg functions. The circulating CD4+CD161+ T cell levels were positively associated with disease activity parameters and autoantibody presence. These results suggest that IL-17-producing CD4+CD161+ T cells might play a role in the inflammation development and B cell activation in pSS.

## Supplementary Material

We compared Hb levels in pSS patients with elevated or normal percentages of CD4+CD161+ T cell subsets. There was no difference in Hb levels between patients with elevated and normal CD4+CD25+CD161+ T cell subset or the overall CD4+CD161+ T cell subset. The Hb level in patients with elevated CD4+CD25-CD161+ subset was significantly higher than that in patients with normal CD4+CD25-CD161+ subset (Table S1). To evaluate the potential function of CD4+CD25+CD161+ T cell subset, we compared the expression of Foxp3 and Helios of CD4+CD25+CD161+ and CD4+CD25+CD161- T cell subsets. It was very obvious that the CD4+CD25+CD161+ T cells expressed significantly decreased levels of Foxp3 or Helios than the CD4+CD25+CD161- T cell subset (Supplementary Fig.1). We further evaluated the expression levels of these two transcription factor of CD4+CD25+CD161+ and CD4+CD25+CD161- T cell subsets in both pSS patients and healthy controls (Supplementary Fig.2). In healthy subjects, the CD4+CD25+CD161+ T cells expressed significantly decreased levels of Foxp3 or Helios than the CD4+CD25+CD161- T cell subset. However, both CD4+CD25+CD161+ and CD4+CD25+CD161- T cell subsets in healthy people expressed less Foxp3 and Helios than their counterparts in pSS patents.

## Figures and Tables

**Table 1:** Clinical and laboratory characteristics in patients with pSS and healthy controls.

Characteristics	pSS	HCs	*p* value
Age	57.84 ± 13.01	51.59 ± 18.58	0.158
Sex (female : male)	56 : 2	15 : 1	0.524
Disease duration (years)	7.53 ± 6.95	NA	
Xerophthalmia (%)	50/58 (86.21)	NA	
Xerostomia (%)	54/58 (93.10)	NA	
ANA(+) (%)	50/58 (86.21)	NA	
Anti-SSA(+) (%)	45/58 (77.59)	NA	
Anti-SSB(+) (%)	19/58 (32.76)	NA	
RF(+) (%)	31/58 (53.45)	NA	
Increased ESR (%)	30/58 (51.72)	NA	
Increased IgG (%)	28/58 (48.28)	NA	
Increased *γ*-globulin (%)	38/58 (65.52)	NA	
ESSDAI	3.86 ± 2.05	NA	

ESSDAI: the EULAR-SS Disease Activity Index; ANA: antinuclear antibody; Anti-SSA: anti-Ro/SSA antibody; anti-SSB: anti-La/SSB antibody; RF: rheumatoid factor; ESR: erythrocyte sedimentation rate; IgG: immunoglobulin A; HCs: healthy controls; NA: not applicable. Numerical data presented as mean ± SD were analyzed using Student's *t*-test or Pearson's Chi-squared test. *p* < 0.05 was taken as significant.

**Table 2:** Clinical and laboratory characteristics of pSS patients with the higher or lower percentages of circulating CD4+CD161+ T cell subsets.

Characteristics	CD4+CD25+CD161+ T (%)		*χ* ^2^	*p* value	CD4+CD25−CD161+ T (%)		*χ* ^2^	*p* value	CD4+CD161+ T (%)		*χ* ^2^	*p* value
	≤6.70%, *n* = 12	>6.70%, *n* = 46			≤17.79%, *n* = 35	>17.79%, *n* = 23			≤17.66%, *n* = 36	>17.66%, *n* = 22		
Xerophthalmia (%)	11 (92)	39 (85)	0.021	0.884	32 (91)	18 (78)	1.068	0.301	32 (89)	18 (82)	0.133	0.715
Xerostomia (%)	12 (100)	42 (91)		0.571	34 (97)	20 (87)	0.937	0.333	35 (97)	19 (86)	1.102	0.294
Parotid gland enlargement (%)	2 (17)	4 (9)	0.076	0.783	4 (11)	2 (9)	0.000	1.000	5 (14)	1 (5)	0.475	0.491
Swollen and/or tender joints (%)	3 (25)	11 (24)	0.000	1.000	8 (23)	6 (26)	0.079	0.779	9 (25)	5 (23)	0.039	0.844
Interstitial lung diseases (%)	4 (33)	15 (33)	0.000	1.000	13 (37)	6 (26)	0.770	0.380	15 (42)	4 (18)	3.419	0.064
Leucopenia (%)	3 (27)	11 (26)	0.000	1.000	8 (25)	6 (27)	0.035	0.851	7 (21)	7 (33)	0.982	0.322
Anaemia (%)	4 (36)	13 (30)	0.001	0.979	8 (25)	9 (41)	1.530	0.216	10 (30)	7 (33)	0.055	0.815
Thrombocytopenia (%)	1 (9)	15 (35)	1.695	0.193	7 (22)	9 (41)	2.265	0.132	7 (21)	9 (43)	2.884	0.089
ANA(+) (%)	9 (75)	41 (89)	0.631	0.427	28 (80)	22 (96)	1.695	0.193	29 (81)	21 (95)	1.450	0.228
Anti-SSA(+) (%)	10 (83)	35 (76)	0.022	0.883	26 (74)	19 (83)	0.553	0.457	28 (78)	17 (77)	0.000	1.000
Anti-SSB(+) (%)	4 (33)	15 (33)	0.000	1.000	11 (31)	8 (35)	0.071	0.790	12 (33)	7 (32)	0.014	0.905
Anti-*α*-Fodrin(+) (%)	0 (0)	2 (4)		1.000	1 (3)	1 (4)		1.000	1 (3)	1 (5)		1.000
RF(+) (%)	7 (58)	24 (52)	0.145	0.703	15 (43)	16 (70)	3.979	**0.046**	16 (44)	15 (68)	3.092	0.079
Decreased C3 (%)	6 (50)	21 (46)	0.072	0.788	8 (23)	8 (35)	0.988	0.320	10 (28)	6 (27)	0.002	0.967
Decreased C4 (%)	1 (8)	13 (28)	1.119	0.290	7 (20)	7 (30)	0.825	0.364	8 (22)	6 (27)	0.190	0.663
Increased ESR (%)	6 (55)	24 (53)	0.005	0.942	12 (36)	18 (78)	9.565	**0.002**	14 (41)	16 (73)	5.346	**0.021**
Increased CRP (%)	0 (0)	5 (11)	0.381	0.537	3 (9)	2 (9)	0.000	1.000	3 (8)	2 (9)	0.000	1.000
Increased IgA (%)	5 (42)	10 (22)	1.069	0.301	9 (26)	6 (26)	0.001	0.975	10 (28)	5 (23)	0.182	0.670
Increased IgG (%)	6 (50)	22 (48)	0.018	0.893	16 (46)	12 (52)	0.232	0.630	18 (50)	10 (45)	0.113	0.737
Increased IgM (%)	1 (8)	4 (9)	0.000	1.000	2 (3)	6 (13)	0.245	0.621	4 (11)	1 (5)	0.146	0.702
Increased *γ*-globulin (%)	8 (67)	30 (65)	0.000	1.000	20 (57)	18 (78)	2.740	0.098	22 (61)	16 (73)	0.816	0.366

pSS: primary Sjögren's syndrome; ANA: antinuclear antibodies; Anti-SSA: anti-Ro/SSA antibody; Anti-SSB: anti-La/SSB antibody; RF: rheumatoid factor; C3: complement component C3; C4: complement component C4; ESR: erythrocyte sedimentation rate; CRP: C-reactive protein; IgA: immunoglobulin A; IgG: immunoglobulin G; IgM: immunoglobulin M (IgM). The values in bold represent ones with statistical significance.

**Table 3:** Correlations of CD4+CD161+ T cell subsets with the laboratory parameters from pSS patients.

Laboratory parameters	CD4+CD25+CD161+ T (%)		CD4+CD25−CD161+ T (%)		CD4+CD161+ T (%)	
	*r*	*p* value	*r*	*p* value	*r*	*p* value
WBC	0.0751	0.5822	0.0235	0.8638	−0.0133	0.9227
Hb	0.0185	0.8926	−0.2458	0.0678	−0.1897	0.1614
PLT	−0.1921	0.1560	−0.2562	0.0566	−0.2736	**0.0413**
Anti-*α*-Fodrin	0.0944	0.4730	−0.0956	0.4677	−0.0909	0.4899
RF	−0.0378	0.7744	0.1313	0.3172	0.1359	0.3003
Anti-SSA	−0.0227	0.8655	0.1826	0.1701	0.1821	0.1713
Anti-SSB	−0.0336	0.8025	0.2562	0.0523	0.2498	0.0586
C3	−0.0562	0.6696	0.0643	0.6257	0.0872	0.5076
C4	−0.1532	0.2426	−0.1012	0.4419	−0.0679	0.6062
ESR	−0.0971	0.4685	0.3145	**0.0162**	0.2776	**0.0349**
CRP	−0.0488	0.7110	0.1824	0.1632	0.1300	0.3222
IgA	−0.1506	0.2509	0.0649	0.6225	0.0908	0.4903
IgG	0.1100	0.4030	0.2139	0.1008	0.2079	0.1109
IgM	−0.0382	0.7720	−0.0326	0.8049	−0.0509	0.6993
*γ*-globulin	0.0999	0.4478	0.2334	0.0727	0.2204	0.0907

pSS: primary Sjögren's syndrome; WBC: white blood cell (leucocyte); Hb: hemoglobin; PLT: platelet; RF: rheumatoid factor; Anti-SSA: anti-Ro/SSA antibody; Anti-SSB: anti-La/SSB antibody; C3: complement component C3; C4: complement component C4; ESR: erythrocyte sedimentation rate; CRP: C-reactive protein; IgA: immunoglobulin A; IgG: immunoglobulin G; IgM: immunoglobulin M (IgM). The values in bold represent results with statistical significance.

**Table 4:** CD4+CD161+T cell subsets in the presence or absence of clinical or laboratory characteristics or ESSDAI ≥ 4 from pSS patients.

Characteristics	CD4+CD25+CD161+T/CD4+CD25+ T (%)		*p* value	CD4+CD25−CD161+T/CD4+CD25− T (%)		*p *value	CD4+CD161+T/CD4+ T (%)		*p* value
	Presence (*n*)	Absence (*n*)		Presence (*n*)	Absence (*n*)		Presence (*n*)	Absence (*n*)	
Xerophthalmia (%)	12.70 ± 7.48 (*n* = 50)	15.19 ± 6.25 (*n* = 8)	0.377	17.47 ± 9.36 (*n* = 50)	19.79 ± 5.33 (*n* = 8)	0.500	17.39 ± 8.92 (*n* = 50)	19.34 ± 5.57 (*n* = 8)	0.553
Xerostomia (%)	12.83 ± 7.39 (*n* = 54)	15.93 ± 6.47 (*n* = 4)	0.419	17.58 ± 9.14 (*n* = 54)	20.65 ± 4.56 (*n* = 4)	0.511	17.47 ± 8.71 (*n* = 54)	20.23 ± 5.42 (*n* = 4)	0.537
Parotid gland enlargement (%)	10.53 ± 4.73 (*n* = 6)	13.33 ± 7.54 (*n* = 52)	0.380	14.27 ± 6.49 (*n* = 6)	18.20 ± 9.11 (*n* = 52)	0.311	13.70 ± 6.51 (*n* = 6)	18.11 ± 8.66 (*n* = 52)	0.233
Swollen and/or tender joints (%)	13.47 ± 9.34 (*n* = 14)	12.91 ± 6.68 (*n* = 44)	0.804	17.73 ± 7.97 (*n* = 14)	17.81 ± 9.28 (*n* = 44)	0.977	17.67 ± 7.82 (*n* = 14)	17.65 ± 8.82 (*n* = 44)	0.994
Interstitial lung diseases (%)	11.13 ± 5.83 (*n* = 19)	13.98 ± 7.85 (*n* = 39)	0.166	15.12 ± 8.33 (*n* = 19)	19.09 ± 9.00 (*n* = 39)	0.111	14.85 ± 8.33 (*n* = 19)	19.03 ± 8.38 (*n* = 39)	0.080
Leucopenia (%)	13.34 ± 7.10 (*n* = 14)	12.28 ± 6.38 (*n* = 40)	0.608	19.88 ± 12.45 (*n* = 14)	17.30 ± 7.64 (*n* = 40)	0.365	19.73 ± 11.49 (*n* = 14)	17.14 ± 7.43 (*n* = 40)	0.338
Anaemia (%)	11.19 ± 5.76 (*n* = 17)	13.18 ± 6.82 (*n* = 37)	0.303	20.95 ± 10.78 (*n* = 17)	16.60 ± 7.96 (*n* = 37)	0.102	20.12 ± 10.17 (*n* = 17)	16.74 ± 7.73 (*n* = 37)	0.183
Thrombocytopenia (%)	14.89 ± 6.01 (*n* = 16)	11.57 ± 6.55 (*n* = 38)	0.087	22.43 ± 11.37 (*n* = 16)	16.09 ± 7.30 (*n* = 38)	**0.018**	22.21 ± 10.69 (*n* = 16)	15.95 ± 6.95 (*n* = 38)	**0.013**
ANA(+) (%)	13.67 ± 7.56 (*n* = 50)	9.10 ± 3.93 (*n* = 8)	0.101	18.64 ± 9.22 (*n* = 50)	12.48 ± 3.86 (*n* = 8)	0.069	18.56 ± 8.71 (*n* = 50)	12.03 ± 4.33 (*n* = 8)	**0.043**
Anti-SSA(+) (%)	12.37 ± 6.61 (*n* = 45)	15.39 ± 9.30 (*n* = 13)	0.193	18.39 ± 9.71 (*n* = 45)	15.72 ± 5.05 (*n* = 13)	0.347	18.21 ± 9.18 (*n* = 45)	15.75 ± 5.57 (*n* = 13)	0.365
Anti-SSB(+) (%)	11.97 ± 5.96 (*n* = 19)	13.57 ± 7.92 (*n* = 39)	0.440	19.15 ± 11.02 (*n* = 19)	17.13 ± 7.76 (*n* = 39)	0.421	18.84 ± 10.40 (*n* = 19)	17.08 ± 7.52 (*n* = 39)	0.465
Anti-*α*-Fodrin(+) (%)	17.70 ± 6.79 (*n* = 2)	12.88 ± 7.34 (*n* = 56)	0.365	16.75 ± 8.13 (*n* = 2)	17.83 ± 9.00 (*n* = 56)	0.868	17.50 ± 8.34 (*n* = 2)	17.66 ± 8.60 (*n* = 56)	0.979
RF(+) (%)	13.23 ± 7.09 (*n* = 31)	12.83 ± 7.71 (*n* = 27)	0.838	19.78 ± 10.39 (*n* = 31)	15.50 ± 6.28 (*n* = 27)	0.060	19.50 ± 9.78 (*n* = 31)	15.54 ± 6.34 (*n* = 27)	0.077
Decreased C3 (%)	14.22 ± 6.89 (*n* = 16)	12.60 ± 7.51 (*n* = 42)	0.455	20.13 ± 12.43 (*n* = 16)	16.90 ± 7.14 (*n* = 42)	0.339	19.93 ± 11.69 (*n* = 16)	16.79 ± 6.94 (*n* = 42)	0.327
Decreased C4 (%)	15.21 ± 6.47 (*n* = 14)	12.36 ± 7.51 (*n* = 44)	0.207	19.45 ± 9.79 (*n* = 14)	17.26 ± 8.67 (*n* = 44)	0.428	19.19 ± 9.20 (*n* = 14)	17.17 ± 8.34 (*n* = 44)	0.444
Increased ESR (%)	12.73 ± 6.86 (*n* = 30)	13.78 ± 8.04 (*n* = 26)	0.600	20.83 ± 9.66 (*n* = 30)	14.74 ± 6.96 (*n* = 26)	**0.010**	20.53 ± 9.35 (*n* = 30)	14.86 ± 6.48 (*n* = 26)	**0.012**
Increased CRP (%)	11.36 ± 2.57 (*n* = 5)	13.20 ± 7.61 (*n* = 53)	0.595	19.82 ± 10.03 (*n* = 5)	17.60 ± 8.88 (*n* = 53)	0.599	18.60 ± 7.83 (*n* = 5)	17.57 ± 8.65 (*n* = 53)	0.798
Increased IgA (%)	11.51 ± 7.51 (*n* = 15)	13.58 ± 7.27 (*n* = 43)	0.350	21.09 ± 12.83 (*n* = 15)	16.64 ± 6.91 (*n* = 43)	0.217	20.65 ± 12.25 (*n* = 15)	16.61 ± 6.65 (*n* = 43)	0.241
Increased IgG (%)	13.67 ± 7.42 (*n* = 28)	12.46 ± 7.31 (*n* = 30)	0.535	19.61 ± 10.38 (*n* = 28)	16.09 ± 7.05 (*n* = 30)	0.135	19.31 ± 9.95 (*n* = 28)	16.11 ± 6.74 (*n* = 30)	0.154
Increased IgM (%)	10.60 ± 4.08 (*n* = 5)	13.27 ± 7.54 (*n* = 53)	0.440	21.78 ± 15.10 (*n* = 5)	17.41 ± 8.24 (*n* = 53)	0.299	20.10 ± 14.15 (*n* = 5)	17.43 ± 7.97 (*n* = 53)	0.507
Increased *γ*-globulin (%)	13.21 ± 6.96 (*n* = 38)	12.74 ± 8.14 (*n* = 20)	0.818	19.54 ± 10.00 (*n* = 38)	14.47 ± 5.07 (*n* = 20)	**0.013**	19.20 ± 9.49 (*n* = 38)	14.73 ± 5.36 (*n* = 20)	**0.026**
ESSDAI ≥ 4	13.13 ± 6.51 (*n* = 31)	12.94 ± 8.28 (*n* = 27)	0.922	20.11 ± 10.37 (*n* = 31)	15.13 ± 6.02 (*n* = 27)	**0.032**	19.71 ± 9.77 (*n* = 31)	15.30 ± 6.18 (*n* = 27)	**0.049**

Data were presented as mean ± SD. pSS: primary Sjögren's syndrome; ANA: antinuclear antibodies; Anti-SSA: anti-Ro/SSA antibody; Anti-SSB: anti-La/SSB antibody; RF: rheumatoid factor; C3: complement component C3; C4: complement component C4; ESR: erythrocyte sedimentation rate; CRP: C-reactive protein; IgA: immunoglobulin A; IgG: immunoglobulin G; IgM: immunoglobulin M (IgM); ESSDAI: the EULAR-SS Disease Activity Index. The values in bold represent ones with statistical significance.

## References

[1] Vitali C., Bombardieri S., Jonsson R. (2002). Classification criteria for Sjögren's syndrome: a revised version of the European criteria proposed by the American-European Consensus Group. *Annals of the Rheumatic Diseases*.

[2] Brito-Zerón P., Ramos-Casals M. (2014). Advances in the understanding and treatment of systemic complications in Sjögren's syndrome. *Current Opinion in Rheumatology*.

[3] Karabiyik A., Peck A. B., Nguyen C. Q. (2013). The important role of T cells and receptor expression in Sjögren’s syndrome. *Scandinavian Journal of Immunology*.

[4] Zhou D., McNamara N. A. (2014). Macrophages: important players in primary Sjögren's syndrome?. *Expert Review of Clinical Immunology*.

[5] van Blokland S. C. A., Wierenga-Wolf A. F., van Helden-Meeuwsen C. G. (2000). Professional antigen presenting cells in minor salivary glands in Sjögren's syndrome: potential contribution to the histopathological diagnosis?. *Laboratory Investigation*.

[6] Adamson T. C., Fox R. I., Frisman D. M., Howell F. V. (1983). Immunohistologic analysis of lymphoid infiltrates in primary Sjogren's syndrome using monoclonal antibodies. *Journal of Immunology*.

[7] Salomonsson S., Jonsson M. V., Skarstein K. (2003). Cellular basis of ectopic germinal center formation and autoantibody production in the target organ of patients with Sjögren’s syndrome. *Arthritis & Rheumatism*.

[8] Cua D. J., Sherlock J., Chen Y. (2003). Interleukin-23 rather than interleukin-12 is the critical cytokine for autoimmune inflammation of the brain. *Nature*.

[9] Nguyen C. G., Yin H., Lee B. H., Carcamo W. C., Chiorini J. A., Peck A. B. (2010). Pathogenic effect of interleukin-17A in induction of Sjögren's syndrome-like disease using adenovirus-mediated gene transfer. *Arthritis Research and Therapy*.

[10] Abdulahad W. H., Boots A. M. H., Kallenberg C. G. M. (2011). FoxP3^+^ CD4^+^ T cells in systemic autoimmune diseases: the delicate balance between true regulatory T cells and effector Th-17 cells. *Rheumatology*.

[11] Lin X., Tian J., Rui K. (2014). The role of T helper 17 cell subsets in Sjögren's syndrome: similarities and differences between mouse model and humans. *Annals of the Rheumatic Diseases*.

[12] Sakai A., Sugawara Y., Kuroishi T., Sasano T., Sugawara S. (2008). Identification of IL-18 and Th17 cells in salivary glands of patients with Sjögren's syndrome, and amplification of IL-17-mediated secretion of inflammatory cytokines from salivary gland cells by IL-18. *Journal of Immunology*.

[13] Katsifis G. E., Rekka S., Moutsopoulos N. M., Pillemer S., Wahl S. M. (2009). Systemic and local interleukin-17 and linked cytokines associated with Sjögren's syndrome immunopathogenesis. *The American Journal of Pathology*.

[14] Pozo D., Valés-Gómez M., Mavaddat N., Williamson S. C., Chisholm S. E., Reyburn H. (2006). CD161 (human NKR-P1A) signaling in NK cells involves the activation of acid sphingomyelinase. *The Journal of Immunology*.

[15] Gulbins E., Kolesnick R. (2003). Raft ceramide in molecular medicine. *Oncogene*.

[16] Cosmi L., De Palma R., Santarlasci V. (2008). Human interleukin 17-producing cells originate from a CD161^+^CD4^+^ T cell precursor. *The Journal of Experimental Medicine*.

[17] Maggi L., Santarlasci V., Capone M. (2010). CD161 is a marker of all human IL-17-producing T-cell subsets and is induced by RORC. *European Journal of Immunology*.

[18] Cosmi L., Cimaz R., Maggi L. (2011). Evidence of the transient nature of the Th17 phenotype of CD4^+^CD161^+^ T cells in the synovial fluid of patients with juvenile idiopathic arthritis. *Arthritis and Rheumatism*.

[19] Miao J., Geng J., Zhang K. (2014). Frequencies of circulating IL-17-producing CD4+CD161+ T cells and CD4+CD161+ T cells correlate with disease activity in rheumatoid arthritis. *Modern Rheumatology*.

[20] Gernez Y., Tirouvanziam R., Nguyen K. D., Herzenberg L. A., Krensky A. M., Nadeau K. C. (2007). Altered phosphorylated signal transducer and activator of transcription profile of CD4^+^CD161^+^ T cells in asthma: modulation by allergic status and oral corticosteroids. *The Journal of Allergy and Clinical Immunology*.

[21] Maggi L., Capone M., Giudici F. (2013). CD4+CD161+ T lymphocytes infiltrate Crohn's disease-associated perianal fistulas and are reduced by anti-TNF-*α* local therapy. *International Archives of Allergy and Immunology*.

[22] Wang T., Sun X., Zhao J. (2015). Regulatory T cells in rheumatoid arthritis showed increased plasticity toward Th17 but retained suppressive function in peripheral bloodis. *Annals of the Rheumatic Diseases*.

[23] Afzali B., Mitchell P. J., Edozie F. C. (2013). CD161 expression characterizes a subpopulation of human regulatory T cells that produces IL-17 in a STAT3-dependent manner. *European Journal of Immunology*.

[24] Dong C. (2008). TH17 cells in development: an updated view of their molecular identity and genetic programming. *Nature Reviews Immunology*.

[25] Maddur M. S., Miossec P., Kaveri S. V., Bayry J. (2012). Th17 cells: biology, pathogenesis of autoimmune and inflammatory diseases, and therapeutic strategies. *The American Journal of Pathology*.

[26] Lin X., Rui K., Deng J. (2015). Th17 cells play a critical role in the development of experimental Sjögren's syndrome. *Annals of the Rheumatic Diseases*.

[27] Ciccia F., Guggino G., Rizzo A. (2012). Potential involvement of IL-22 and IL-22-producing cells in the inflamed salivary glands of patients with Sjögren's syndrome. *Annals of the Rheumatic Diseases*.

[28] Fei Y., Zhang W., Lin D. (2014). Clinical parameter and Th17 related to lymphocytes infiltrating degree of labial salivary gland in primary Sjögren's syndrome. *Clinical Rheumatology*.

[29] Brayer J. B., Cha S., Nagashima H. (2001). IL-4-dependent effector phase in autoimmune exocrinopathy as defined by the NOD.IL-4-gene knockout mouse model of Sjögren's syndrome. *Scandinavian Journal of Immunology*.

[30] Gao J., Killedar S., Cornelius J. G., Nguyen C., Cha S., Peck A. B. (2006). Sjögren's syndrome in the NOD mouse model is an interleukin-4 time-dependent, antibody isotype-specific autoimmune disease. *Journal of Autoimmunity*.

[31] Nguyen C. Q., Gao J.-H., Kim H., Saban D. R., Cornelius J. G., Peck A. B. (2007). IL-4-STAT6 signal transduction-dependent induction of the clinical phase of Sjögren's syndrome-like disease of the nonobese diabetic mouse. *Journal of Immunology*.

[32] Gao J., Cha S., Jonsson R., Opalko J., Peck A. B. (2004). Detection of anti-type 3 muscarinic acetylcholine receptor autoantibodies in the sera of Sjögren's syndrome patients by use of a transfected cell line assay. *Arthritis & Rheumatism*.

[33] Gliozzi M., Greenwell-Wild T., Jin W. (2013). A link between interferon and augmented plasmin generation in exocrine gland damage in Sjögren's syndrome. *Journal of Autoimmunity*.

[34] Zhang X., Schaumburg C. S., Coursey T. G. (2014). CD8^+^ cells regulate the T helper-17 response in an experimental murine model of sjögren syndrome. *Mucosal Immunology*.

[35] Lavoie T. N., Stewart C. M., Berg K. M., Li Y., Nguyen C. Q. (2011). Expression of Interleukin-22 in Sjögren's syndrome: significant correlation with disease parameters. *Scandinavian Journal of Immunology*.

[36] Mitsdoerffer M., Lee Y., Jäger A. (2010). Proinflammatory T helper type 17 cells are effective B-cell helpers. *Proceedings of the National Academy of Sciences of the United States of America*.

[37] Alunno A., Carubbi F., Caterbi S. (2014). The role of T helper 17 cell subsets in Sjögren's syndrome: similarities and differences between mouse model and humans. *Annals of the Rheumatic Diseases*.

[38] Vincent F. B., Saulep-Easton D., Figgett W. A., Fairfax K. A., Mackay F. (2013). The BAFF/APRIL system: emerging functions beyond B cell biology and autoimmunity. *Cytokine and Growth Factor Reviews*.

[39] Deng F., Chen J., Zheng J. (2015). Association of BAFF and IL-17A with subphenotypes of primary Sjögren's syndrome. *International Journal of Rheumatic Diseases*.

[40] González-Hernández Y., Pedraza-Sánchez S., Blandón-Vijil V. (2007). Peripheral blood CD161^+^ T cells from asthmatic patients are activated during asthma attack and predominantly produce IFN-*γ*. *Scandinavian Journal of Immunology*.

[41] Kang K. Y., Kim H.-O., Kwok S.-K. (2011). Impact of interleukin-21 in the pathogenesis of primary Sjögren's syndrome: increased serum levels of interleukin-21 and its expression in the labial salivary glands.. *Arthritis Research & Therapy*.

[42] Poggi A., Costa P., Zocchi M. R., Moretta L. (1997). NKRP1A molecule is involved in transendothelial migration of CD4^+^ human T lymphocytes. *Immunology Letters*.

[43] Campbell J. J., Brightling C. E., Symon F. A. (2001). Expression of chemokine receptors by lung T cells from normal and asthmatic subjects. *The Journal of Immunology*.

[44] Basdeo S. A., Moran B., Cluxton D. (2015). Polyfunctional, pathogenic CD161^+^ Th17 lineage cells are resistant to regulatory T cell-mediated suppression in the context of autoimmunity. *The Journal of Immunology*.

[45] Liu M.-F., Lin L.-H., Weng C.-T., Weng M.-Y. (2008). Decreased CD4+CD25+bright T cells in peripheral blood of patients with primary Sjögren's syndrome. *Lupus*.

