# Supplementary material for: The Clinical Relevance of IL-17-Producing CD4+CD161+ Cell and Its Subpopulations in Primary Sjögren's Syndrome
Source: J Immunol Res. 2015 Sep 8;2015:307453. doi: 10.1155/2015/307453 (PMC4578753; doi:10.1155/2015/307453)
Supplement: Supplementary file 1 — We compared Hb levels in pSS patients with elevated or normal percentages of CD4+CD161+ T cell subsets. There was no difference in Hb levels between patients with elevated and normal CD4+CD25+CD161+ T cell subset or the overall CD4+CD161+ T cell subset. The Hb level in patients with elevated CD4+CD25-CD161+ subset was significantly higher than that in patients with normal CD4+CD25-CD161+ subset (Table S1). To evaluate the potential function of CD4+CD25+CD161+ T cell subset, we compared the expression of Foxp3 and Helios of CD4+CD25+CD161+ and CD4+CD25+CD161- T cell subsets. It was very obvious that the CD4+CD25+CD161+ T cells expressed significantly decreased levels of Foxp3 or Helios than the CD4+CD25+CD161- T cell subset (Supplementary Fig.1). We further evaluated the expression levels of these two transcription factor of CD4+CD25+CD161+ and CD4+CD25+CD161- T cell subsets in both pSS patients and healthy controls (Supplementary Fig.2). In healthy subjects, the CD4+CD25+CD161+ T cells expressed significantly decreased levels of Foxp3 or Helios than the CD4+CD25+CD161- T cell subset. However, both CD4+CD25+CD161+ and CD4+CD25+CD161- T cell subsets in healthy people expressed less Foxp3 and Helios than their counterparts in pSS patents. [file 307453.f1.pdf]

Supplementary Data

Table S1 Hb of pSS patients with the elevated or normal percentages of circulating CD4+CD161+ T cell subsets.

Character-istics	CD4+CD25+ CD161+T (%)		*p* value	CD4+CD25- CD161+T (%)		*p* value	CD4+CD161+T (%)		*p* value
	≤6.70	>6.70		≤17.79	>17.79		≤17.66	>17.66	
Hb	128.5± 19.98	119.3± 15.96	0.2780	124.7± 17.48	116.2± 15.43	0.0428	122.1± 19.57	119.8± 12.41	0.3422

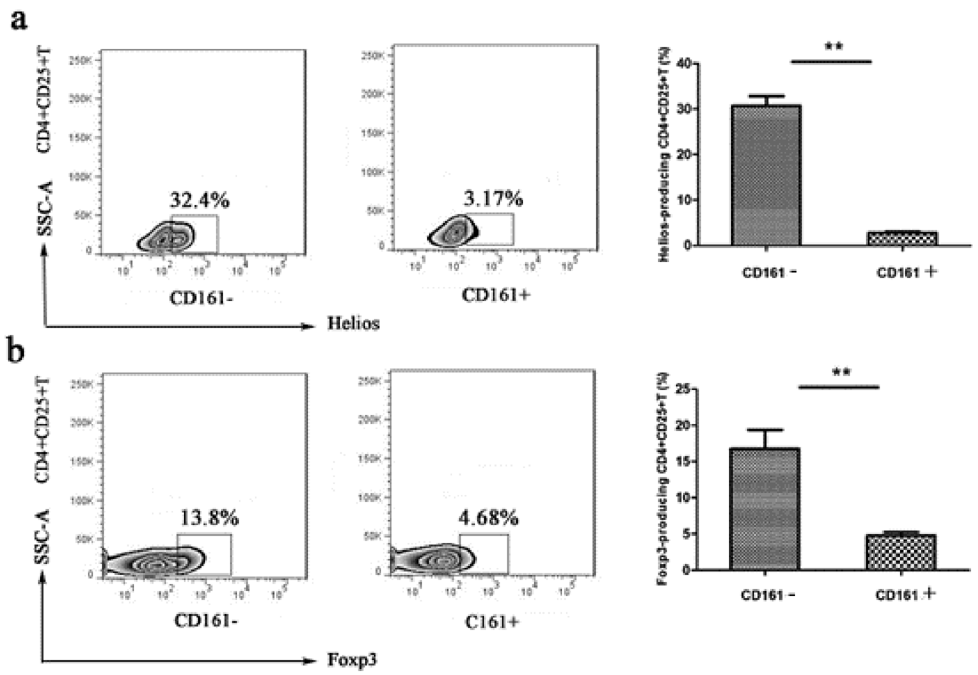

Supplementary Fig.1 Foxp3 and Helios expression of the CD4+CD25+ CD161+ T cell subsets in HCs. Representative flow cytometric plots of transcription factors production in CD161+ or CD161- T cell subsets were shown. Frequencies of Foxp3-producing or Helios-producing cells in CD161+ and CD161- fractions in HCs (n=5) were compared. ***p*<0.01

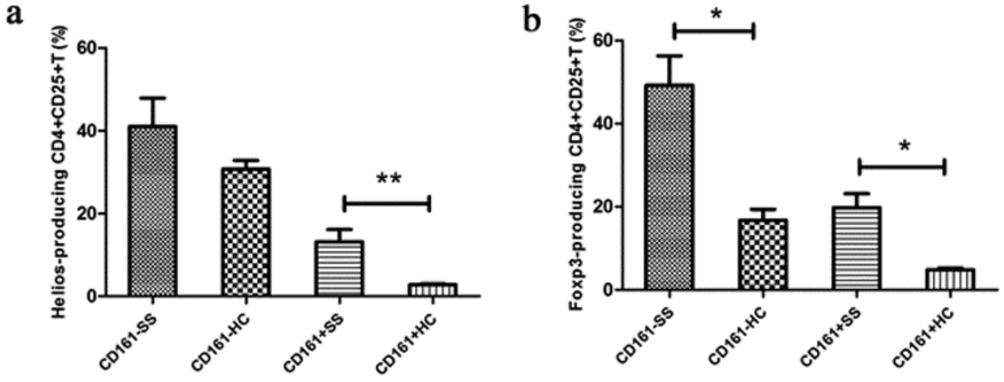

Supplementary Fig.2 Foxp3 and Helios expression of the CD4+CD25+ CD161+ T cell subsets in pSS and HCs. Percentages of Foxp3 producing or Helios producing cells in CD161+ and CD161- fractions in HCs (n=5) were compared. *$p<0.05$, ** $p<0.01$.
